# Lentiviral Vectors: From Wild-Type Viruses to Efficient Multi-Functional Delivery Vectors

**DOI:** 10.3390/ijms26178497

**Published:** 2025-09-01

**Authors:** Ane Arrasate, Carlos Lopez-Robles, Miren Zuazo, Soledad Banos-Mateos, Cesar Martin, Andrés Lamsfus-Calle, Marie J. Fertin

**Affiliations:** 1 VIVEbiotech, 20014 Donostia-San Sebastian, Spain; clopez@vivebiotech.com (C.L.-R.); mzuazo@vivebiotech.com (M.Z.); aarrasate@vivebiotech.com (A.A.); mfertin@vivebiotech.com (M.J.F.); 2 Department of Biochemistry and Molecular Biology, University of the Basque Country UPV/EHU, 48940 Bilbao, Spain; cesar.martin@ehu.eus; 3 Biofisika Institute (UPV/EHU, CSIC), Barrio Sarriena s/n., 48940 Leioa, Spain

**Keywords:** lentiviral vectors, HIV-1, LVV manufacturing, CAR-T, in vivo gene therapy, ex vivo gene therapy, RNA delivery, vaccinology

## Abstract

Extensive studies about the human immunodeficiency virus type 1 (HIV-1) have allowed the generation of lentiviral vectors as gene delivery vehicles with enhanced safety and efficacy features. In this review, several strategies for controlling the molecular mechanisms occurring during the lentiviral vector manufacturing process are presented. Specifically, modifications focused on LVV manufacturing components, such as plasmids or the producer cell line, that enable increased safety, integrity, and potency of the produced LVV, as well as manufacturing efficiency. Considering the stochasticity of the LVV manufacturing process from plasmid transfection until the budding of the virus from the target cell, minimal modifications might have a huge impact on the final LVV yield. Indeed, the extent of a potential impact may vary depending on the specificities of each LVV regarding the particular genetic payload or the envelope protein. Thus, the feasibility of each of the optimizations described herein requires thorough evaluation. The second part of the review examines the potential multi-purpose nature of the LVV. Growing research in the field has enabled the development of new engineered modalities of LVV, expanding their application scope beyond the traditional ex vivo DNA delivery approach. LVVs are becoming a versatile tool for the packaging or delivery of cargo in the form of DNA, RNA, or protein, allowing their use for in vivo approaches, vaccinology, or gene editing, among others.

## 1. Introduction

### 1.1. Overview of Lentiviruses Origin

**Figure 1:**
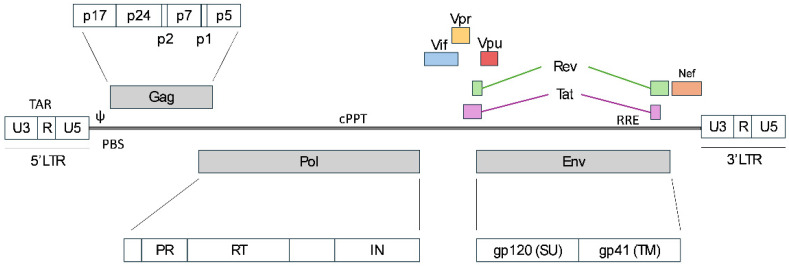
Schematic illustration of the HIV-1 genome.

Lentiviruses, a genus within the *Retroviridae* family, include human immunodeficiency virus type 1 (HIV-1) as their most well-characterized member. Current lentiviral vectors (LVVs) have primarily been derived from HIV-1 through extensive research and molecular engineering for maximized safety without compromising functionality [1]. HIV-1 is characterized by its genome (Figure 1), which comprises two copies of single-stranded, positive-sense RNA [2]. Each strand is flanked by long terminal repeats (LTRs) at both ends, which are essential for transcription, reverse transcription, and integration of the viral genome. Each LTR contains three elements: U3, R, and U5. The U3 element acts as a promoter/enhancer in the 3′LTR, while the R element serves as a polyadenylation signal in the 5′LTR. Thus, provirus mRNA is devoid of 5′ U3 and 3′ U5, resulting in a viral RNA flanked by R elements [3]. In addition, the U5 element in the 5′ end is part of a complex secondary structure that modulates key steps like packaging or reverse transcription [4]. The genome encodes nine proteins through nine Open Reading Frames (ORFs), some of which are proteolytically processed to generate additional proteins. Among these, the two primary proteins are the Gag/Pol polyprotein and the Env glycoprotein. *Gag* expresses the viral core proteins, which are matrix protein (MA or p17), capsid (CA or p24), nucleocapsid (NC or p7), and smaller core proteins p6, p1, and p2. *Pol* translates into the viral enzymes protease (PR) for proteolytic processing of Gag and Gag-Pol polyproteins, the reverse transcriptase (RT), which has both DNA polymerase and RNase H activity, and the integrase (IN) for viral genome integration into the host cell. In addition, the *Env* gene encodes for the viral surface glycoprotein (SU or gp120) as well as the transmembrane glycoprotein (TM or gp41). Additionally, the viral genome encodes the regulatory protein Tat (trans-activator of transcription) for transcription activation and Rev (Regulator of Expression of Virion proteins) for splicing control and nuclear export. The remaining genes are responsible for encoding the accessory proteins Vif (Virion Infectivity Factor), Vpr (Viral Protein R), Vpu (Viral Protein U), and Nef (Negative Factor) [3,5].

In addition to those coding genes, several elements are present in the lentivirus genome for its life cycle. The HIV-1 genome transcript undergoes many splicing events between its four donor and ten acceptor sites to create different transcripts that will lead to the proteins required for virus formation [6]. Initial transcription in the absence of Tat uniquely expresses the totally spliced short transcripts *Tat*, *Rev*, and *Nef*. Once synthesized, Tat interacts with the transactivation-response element (TAR) located on the R of the LTR and induces the transcription of unspliced or partially spliced mRNAs. The cellular mRNA mechanism does not permit the export of mRNAs containing introns from the nucleus to the cytoplasm; nevertheless, this transport is facilitated by the Rev protein, which binds to RRE sequences located at those mRNAs [6]. Moreover, to ensure packaging of the full-length RNA, this transcript contains the packaging signal (ψ) downstream of the 5′LTR. Other elements, such as the primer binding site (PBS) and the polypurine tract (PPT), allow the initiation of the viral genome reverse transcription into double-stranded DNA (dsDNA) for subsequent integration into the host cell genome [7].

### 1.2. General Virological Properties of Lentiviruses

**Figure 2:**
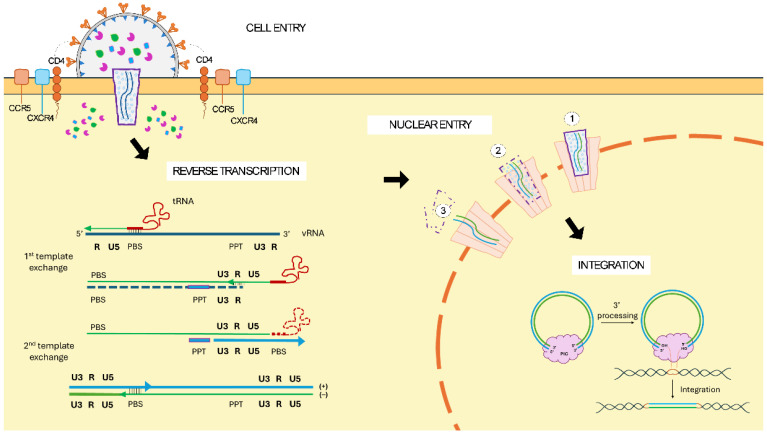
Schematic illustration of cellular entry, reverse transcription, nuclear entry models and genome integration.

The transduction process of the target cell is mediated by the interaction of the proteins within the viral envelope with the specific cell membrane receptor (Figure 2). Wild-type HIV-1 cell entry is mediated by two envelope proteins, gp120 for receptor binding and gp41 for membrane fusion, which together form a labile heterodimer (gp160). The trimeric configuration of those heterodimers forms the viral spike that interacts with the CD4 receptor in target cells. Several conformational changes enable the binding of gp120 to the coreceptors CCR5 or CXCR4, followed by gp41 translocation into the cell membrane that results in viral core entry to the cytoplasm [8]. Thus, HIV-1 primarily infects activated T cells via the CD4 receptor and CCR5 coreceptor, and additionally naïve CD4+ T cells through the CXCR4 coreceptor, as well as macrophages, despite their lower levels of CD4 [9].

Following entry, the reverse transcriptase protein (RT) initiates the reverse-transcription process of the viral RNA genome (Figure 2). Lentiviruses package two RNA copies of the complete genome into a single virion in a dimeric form, which allows switching templates during reverse transcription in case of RNA damage, ensuring genome integrity [10]. The reverse transcription from an initial viral positive-sense single-stranded RNA (+ssRNA) molecule is a complex process that includes two template exchange steps resulting in the synthesis of a double-stranded DNA. The process starts from the 5′ end of the RNA molecule with the synthesis of the (-) DNA strand. Important elements involved in this process include the PBS present in the 5′ end of the RNA and the annealing transfer RNA (tRNA^Lys3^) molecule for initiation. The first template exchange allows the polymerization to continue at the 3′ end of the viral RNA due to the complementarity of its R element and the one in the newly synthesized DNA molecule. Simultaneously, the RNA template is degraded by the RNase H activity of the reverse transcriptase. Only the PPT element is not degraded, which functions as a primer binding site for the synthesis of the (+) strand DNA. Finally, there is a second template exchange through PBS elements complementarity between the partial DNA strands that leads to complete dsDNA synthesis. Ultimately, the final product is a linear DNA duplex copy of the viral genome with the LTRs at either end, containing critical cis-acting signals [7]. Subsequently, dsDNA molecules resulting from reverse transcription potentially exist as double-stranded linear molecules or circular molecules with a single or two LTR elements [11], of which the linear dsDNA is the substrate for host cell genome integration.

The sequence of events widely assumed is that viral uncoating happens upon cell transduction, followed by reverse transcription and subsequent nuclear entry for integration. The nuclear entry of lentiviruses is an active import mechanism mediated by specific nucleoporins and importins that constitute nuclear pore complexes (NPC), which allows the lentivirus to transduce both dividing and non-dividing cells [12,13]. However, the mechanism of this entry is not well described, as experimental evidence proposes different versions (Figure 2). The fact that lentiviral capsids are larger than the diameter of the central channel of NPCs supported the theory of the early uncoating right after cell transduction. Nevertheless, conflicting experimental evidence suggests that capsid uncoating might occur otherwise. In fact, several studies suggest that a stable capsid is required for efficient reverse transcription and viral infection. An alternative model suggests that capsid uncoating initiates when the virus is docked in the NPC, which was identified through live-cell single-virus imaging studies. Recently, cryo-electron tomography (CryoET) analysis revealed that intact or nearly intact HIV-1 cores penetrate through the NPCs, and uncoating occurs inside the nucleus, near integration sites. Therefore, we could assume that reverse transcription occurs inside the viral capsid prior to its release [14]. Nevertheless, the mechanism of NPC crossing, the interacting host cell factors, or the sequence of the steps still needs to be clarified.

Regardless of the nuclear entry mechanism, the integration process is mediated by the pre-integration complex (PIC), whose major functional component is the IN protein, a specialized DNA recombinase (Figure 2). Within the PIC, the ends of the linear viral reverse transcriptase are held together by a multimer of integrase in a complex called an intasome that promotes DNA recombination. For integration to happen, the integrase processes the 3′ end of the DNA molecules, and the resulting 3′-hydroxil groups act as nucleophiles to cleave the host cell genome. The resulting integrated DNA is then flanked by two 4–6 base pair duplications due to cell repairing mechanisms [12,15]. Lentiviruses naturally integrate their genome into actively transcribed genes; thus, their integration pattern is determined by the specific transcriptional program of the target cell [16].

### 1.3. The Evolution from HIV-1 to 3rd Generation LVV

The development of lentiviruses as gene delivery vectors evolved through several generations, each incorporating enhanced safety features. To address the safety considerations, the HIV-1 genome was progressively separated into several plasmids, which largely abolished the probability of replication-competent lentivirus (RCL) generation. This way, the first-generation LVV system is constituted by three plasmids: the packaging plasmid comprising Gag-Pol and regulatory/accessory proteins, the envelope plasmid for vector–cell interaction, and the transfer genome [3]. The packaging plasmid and the envelope plasmids are expressed by a heterologous promoter and do not contain packaging signals or LTRs. Furthermore, instead of the HIV-1 envelope, lentiviral vectors are commonly pseudotyped by the heterologous VSV-G (vesicular stomatitis virus glycoprotein), which provides higher stability during the lentiviral vector production and broad tropism to the vector. Thus, the transduction mechanism of the viral vector is modified from wild-type HIV-1, as VSV-G-mediated cellular entry is enabled through interaction with the ubiquitous receptor LDL-R (low-density lipoprotein receptor) in the target cell membrane. Subsequently, VSV-G undergoes a clathrin-dependent endocytosis process in which actin is also required [17,18,19,20]. The virus is trafficked through the endocytic pathway with a progressively lower pH, leading to fusion of the endosomal and viral membranes, to finally release the capsid into the cell [17,18,19,20]. Finally, the transfer plasmid contains an internal promoter for the expression of the transgene in the transduced cells, since the promoter activity of the 5′LTR is weak in the absence of Tat [3].

Next, second-generation LVV incorporates additional modifications into those plasmids [3]. For instance, the accessory proteins Vif, Vpu, Vpr, and Nef are removed as their primary role involves interactions with host factors permitting virulence and pathogenicity. These proteins are devoid of enzymatic activity, and their presence has been demonstrated to be dispensable for lentiviral vector production, as their removal does not inhibit the transfer of genetic material to the host cell [21,22,23]. Similarly to the first generation, the transfer plasmid is transcribed from a complete 5′LTR. But, for additional safety, self-inactivating (SIN) lentiviral vectors were developed by introducing a deletion in the 3′ LTR U3 region, hence eliminating sequences encoding enhancer and promoter functions. During the reverse transcription process, the U3 region from the 3′LTR serves as a template for 5′LTR reconstitution, thus transferring the deletion of SIN 3′LTR to 5′LTR. This modification further secured LVV-based gene therapies by inactivating potentially packageable viral genome transcription after reverse transcription and integration [24].

**Figure 3:**
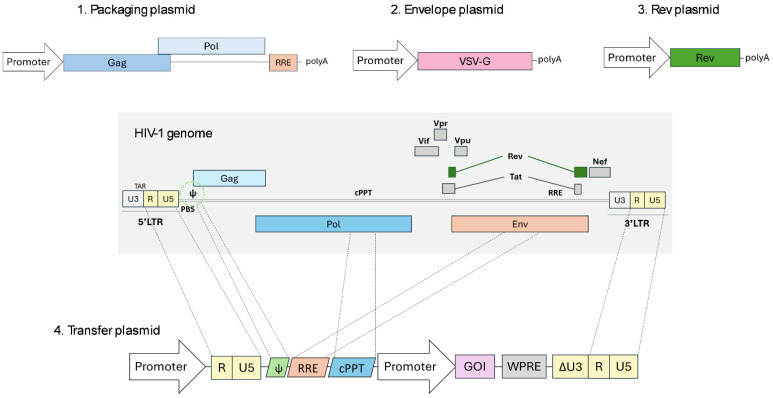
Schematic figure of third-generation lentiviral vectors derived from the HIV-1 genome. The wild-type HIV-1 genome in the center of the figure serves to compare the reduction in the viral elements within the transfer plasmid, as well as to show the distribution of the helper elements across the separate plasmids.

Currently, the third-generation LVV system is the most frequently utilized and is characterized by further dividing the system into a four-plasmid configuration (Figure 3) [3]. The first one is the packaging plasmid that encodes the Gag-Pol polyprotein. Secondly, the envelope plasmid usually encodes the heterologous VSV-G, as previously mentioned. Thirdly, the Rev encoding gene was separated into an additional plasmid responsible for nuclear export of unspliced or partially spliced transcripts. Finally, the transfer plasmid transcribes the viral genome and contains the gene of interest, along with the essential viral cis-acting elements such as the LTRs, packaging signal, or RRE required for viral RNA transport. For increased safety, the replacement of the 5′LTR U3 promoter/enhancer by a strong promoter such as CMV conferred Tat independence to the vector, which allowed the elimination of an additional viral element with no other impacts on lentiviral vector functioning [25,26].

Whilst the primary focus of the initial modifications was an increased safety profile, additional cis-acting elements were also introduced to the transfer genome, aiming to boost the efficiency of LVVs as delivery vectors. For instance, the insertion of a cPPT sequence (derived from pol) in the transfer genome enhances the transduction efficiency, as it serves as the second initiation site during reverse transcription [27]. Additionally, the inclusion of the post-transcriptional regulatory element of the woodchuck hepatitis virus (WPRE) at the 3′ untranslated region of the lentiviral transgenes improves viral titer and transgene expression due to an improvement in transcription termination and an increase in polyadenylated transcript concentration [28,29,30,31,32]. Safety concerns arose because its sequence contains part of the coding reading frame of the potentially oncogenic woodchuck hepatitis virus X protein (WHX) [33]. Therefore, alternative WPRE variants have emerged, introducing single mutations into the promoter and the start codon of the WHX ORF or even deleting the complete promoter sequence to prevent any potential expression of truncated WHX protein and derived peptides [30,34].

### 1.4. Current Situation of Lentiviral Vector in Gene Therapy and Research

Lentiviral vectors are successful delivery tools for gene and cell therapy due to their unique biological features. These vectors offer a broad range of target cell transduction of both dividing and non-dividing cells, a large packaging capacity, and an efficient integration into the host cell genome, allowing a long and stable transgene expression [1]. Currently, LVVs are mainly utilized for ex vivo modification of target cells in cell therapy, which provides a controlled gene delivery. This method is based on target cell extraction from the patient and subsequent ex vivo transduction with LVVs prior to reinfusion into the same individual (autologous transplant) or a different patient (allogeneic transplant) [35,36]. Accordingly, the entirety of LVV-based therapies for commercial use so far are based on ex vivo transduction of human blood cells. A subset of those therapies involves chimeric antigen receptor (CAR)-T cells, where autologous T cells are transduced with specific CAR-expressing SIN-LVV, all aimed at specific cancer treatments such as multiple myeloma or large B-cell lymphoma [37]. Indeed, those engineered CAR-T cells are modified to recognize and attack cancer cells when the encoded chimeric T cell receptor binds to a specific antigen on its surface. The remaining ones are focused on restoring the deficient genes associated generally with rare diseases by integrating a biologically active copy into hematopoietic stem cells (HSCs) [37]. Among them, Lenmeldy is based on ARSA gene delivery for the treatment of metachromatic leukodystrophy (MLD); Skysona on transferring adrenoleukodystrophy protein (ALDP) encoded by the ABCD1 gene to treat cerebral adrenoleukodystrophy (CALD); and Lyfgenia and Zynteglo for the treatment of both sickle cell disease and β-thalassemia by providing functional copies of a modified β^A^-globin gene, respectively [37].

The outcome of those therapies strongly relies on the efficiency and safety of the LVV, which highlights the importance of their manufacturing process and characterization. Thus, this review focuses on optimizations performed in the current LVV production system, putting the spotlight on the LVV gene constructs and the producer cell line. Furthermore, lentiviral vectors are taking an increasingly prominent role in new application fields, such as vaccines and gene editing therapies, as a result of novel and innovative developments in this gene delivery platform. Detailed insights into this regard are also mentioned in this article.

## 2. State of the Art of Manufacturing

**Figure 4:**
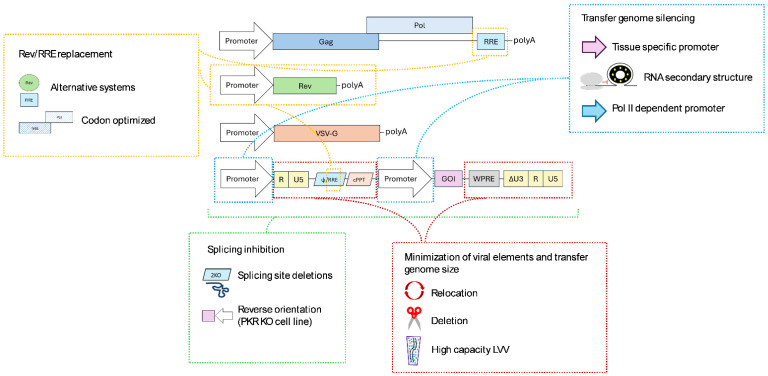
Schematic illustration of the potential optimizations in the manufacturing process of lentiviral vectors. The four plasmids comprising the third-generation LVV are displayed, with the modifications listed alongside, indicating the specific plasmid to which each applies.

The exponential growth of the lentiviral vector specialized industry is driving Contract Development and Manufacturing Organizations (CDMOs) to constantly and rapidly adapt to the requirements of the drug developers and the regulatory agencies for safer, cost-efficient, and reproducible manufacturing processes. Regulatory agencies dictate the requirements for clinical and commercial use of lentiviral vectors, which cover quality, safety, efficacy, and manufacturing and biological aspects of the therapy. Specific considerations about LVV-based therapies include the determination of gene expression persistence or risk of insertional mutagenesis or RCL generation, among others. Ultimately, preclinical data should provide sufficient information to allow a proper risk assessment for the use of the advanced therapy medicinal product (ATMP) in human subjects. Thus, variations in sequences within the lentiviral construct, including the promoter, gene of interest, or any regulatory element, should be thoroughly evaluated, providing regulators with evidence about the impact of potential changes in therapeutic product quality [38]. Accordingly, it might be logical to expect that regulatory demands will become more specific and evidence-based as the field continues to grow rapidly; hence, identifying areas for optimization is essential to meet future demands. To this end, an in-depth comprehension of the biological mechanisms involved in LVV production facilitates the identification of critical optimization targets within the lentiviral plasmid constructs and the producer cell line. Altogether, batches with enhanced potency and purity will contribute to more efficient transduction of target cells with reduced adverse effects. In the following lines, a detailed discussion of those optimizations will be further reviewed (Figure 4).

### 2.1. Optimizations in Lentiviral Constructs

The third-generation LVV system reduced the initial nine proteins from HIV-1 into three genes (Gag, Pol, and Rev) by the removal of all the accessory genes and replacement of the HIV-1 envelope protein. However, the multi-plasmid transfection system has inherent molecular biology challenges, primarily associated with the transfer plasmid. Following, the optimization strategies developed for an efficient production process with enhanced potency, integrity, and safety of LVV are discussed.

#### 2.1.1. Rev/RRE System Replacement

For optimal vector production, Rev presence is necessary for its interaction with the *Rev* responsive element (RRE), leading to efficient nuclear export of unspliced Gag-Pol and vector genome RNA transcripts. Considering cost-efficiency and safety, different attempts to develop *Rev*-independent production systems have been proposed to reduce the total number of transfected plasmids and minimize homologous sequences between them that may promote potential recombination, respectively.

Initial strategies centered on the identification of heterologous viral elements for the export and stability of unspliced transgene transcripts for the replacement of the Rev/RRE system [39]. To overcome the trans-acting nuclear export mechanism used by complex viruses such as HIV-1, several cis-acting elements were identified as responsible for nuclear export in simpler viruses. For instance, the constitutive transport element (CTE) from Mason-Pfizer monkey virus (MPMV) or, similarly, simian retrovirus type 1 (SRV-1) can fulfill this cis-acting function; however, produced viral titers were considerably lower [40,41]. Additional experiments demonstrated that four consecutive copies of CTE promoted protein expression better than one copy, although RRE was still more efficient for lentiviral vector production [42]. Alternatively, the mammalian genome-derived RNA transport element (RTE) was identified, which allowed an efficient export of transcripts in RRE and rev-defective production systems [43]. Another strategy is based on the identification of a cellular counterpart that replaces the *rev* function. In these lines, a Rev cellular equivalent was identified, Sam68 (Src binding protein in mitosis), that interacted with RRE and could partially substitute Rev as a post-transcriptional regulator [44].

Besides nuclear export, Rev might have a role in the stability of RNA molecules [45], as well as a positive role in protein translation [46,47]. In fact, Rev dependency of the Gag-Pol gene may be attributed to the presence of RNA instability sequences within its transcript. This instability is linked to the numerous AU-rich regions present in Gag-Pol, which additionally implies an extreme codon bias for its expression in mammalian cells [48,49]. Therefore, several groups have developed synthetic Gag-Pol sequences by modifying the nucleotide sequence to replace the AU-rich regions and provide better stability, along with the aim to establish favored codon usage for human cells. Hence, this synthetic Gag-Pol is Rev-independent, so RRE might be removed [48,50,51]. This way, Rev is only required for vector genome nuclear export, and the homology regions between the plasmids are reduced.

#### 2.1.2. Minimization of Viral Elements and Transfer Genome Size

Reducing the content of viral elements from the vector genome is a crucial strategy for enhancing the safety of the vectors and additionally to minimize the size of the packageable genome, as there is substantial evidence indicating that viral titers decrease semi-logarithmically with increasing vector length [52,53]. Lentiviral vectors are capable of packaging vector genomes with approximately 10 kilobases (kb), from which around 1.5 kb belong to HIV-1 sequences. As previously mentioned, these viral elements include several cis-acting elements for RNA processing and packaging into viral particles, comprising the LTR at each end, marking the RNA that is going to be reverse transcribed, along with some substantial regions of Gag and Env [54].

One of the strategies to reduce the presence of viral elements within the vector involves the LTR1 lentiviral vectors [55]. This novel vector removed 5′ R and U5 and relocated the psi and RREs downstream of the SIN 3′LTR. This approach ensures efficient RNA packaging and processing during lentiviral production by preserving the necessary HIV-1 structures, while excluding these elements after reverse transcription, thereby preventing their integration into the target cell genome. An additional PBS element located downstream of the 3′LTR allows reverse transcription mediated by a single-strand transfer instead of the usual two, which has been demonstrated to accelerate transgene expression over standard lentiviral configuration. Therefore, LTR1 vectors allow for improved safety by minimizing viral elements integrated into the host cell genome, while maintaining transgene expression efficiency. As a counterpoint, the production of these vectors is performed with Tat, hence introducing viral elements that were already removed in third-generation LVVs [55].

Following this objective, a study was conducted to evaluate the functional role of *Gag* and *Env* sequences present in the vector genome to determine the essential parts [54]. A systematic deletion analysis concluded that 850 nucleotides of HIV-1 *Gag* and *Env* sequences might be removed from the vector genome without compromising transduction efficiency and vector titer. The RRE sequence constituted the essential region from *Env*, which was shown to be required for optimal vector titer and transgene expression. Furthermore, given the presence of splicing signals (donor and acceptor sites) within the vector genome, the impact of the deletions on the splicing pattern of the vector genome was analyzed. The sequencing results showed that splicing acceptor 7 (SA7) removal within the *Env* deletions increased the unspliced RNA transcripts, although this did not translate into a higher vector yield. They also tried the RRE displacement downstream of the 3′ end combined with a 343-nucleotide deletion in gag without substantially compromising vector titer. This study demonstrated the feasibility of reducing viral elements and, therefore, the size of the vector genome by removing non-essential parts. However, the specific splicing profile is expected to vary depending on the transgene cassette; hence, the deletion application needs to be evaluated for each specific transfer genome construct to determine whether a benefit is achieved [54].

Also, additional strategies have been employed to overcome constraints related to genome size, which continues to pose a struggle for large gene packaging as well as for designing multiple gene-correcting therapies. Recently, a new technology has been applied for a patent (WO2023212396), called high capacity LVV [56]. It is well established that each lentiviral particle packages a dimer of RNA molecules, which leads to the packaging of a pseudo-diploid genome into assembling virions. This dimerization mechanism is performed by interactions between GC-rich residues present in a region called the Dimerization Initiation Site (DIS), and additionally, the *gag* gene start codon (AUG) and the upstream U5 element are indirectly involved [57,58,59]. Each viral particle includes two identical RNA molecules (homodimers), although it has been identified that heterodimer formation is also possible [60,61]. The high-capacity LVVs are based on modifications in specific dimerization-involved sequences to favor the heterodimer formation and packaging, thus allowing for an increase in the genetic payload. This novel approach to increasing the cargo capacity may enable larger or multiple therapeutic gene packaging [56]. However, further investigations are essential to assess the practical advantages of this technology.

#### 2.1.3. Transfer Genome Silencing

Lentiviral transgenes currently used in gene therapies generally feature a strong promoter for constitutive and ubiquitous expression in order to provide a high expression of the therapeutic gene, which potentially guarantees treatment efficacy. However, this strategy may lead to reduced lentiviral production efficiency due to the potential cytotoxicity of the therapeutic gene expression in the producer cell line. The expression of the gene of interest (GOI) might happen due to aberrant splicing events that lead to external promoter-mediated transcription, or, alternatively, through transcripts starting from the internal heterologous promoter. Furthermore, unintended CAR expression in the producer cell might lead to its presence in the LVV membrane and thus off-target transduction via CAR-antigen binding to cells [62]. One of the strategies that might avoid these phenomena is to express the GOI with a tissue-specific promoter. This way, the GOI is silenced during lentiviral production, and after transduction, the GOI is expressed in a more physiological manner in comparison to strong constitutive promoters. Nonetheless, GOI expression can still be related to splicing events occurring from the transcription coming from the external promoter.

Additionally, a transgene repression system was developed to enable silencing during vector production [63]. This system employs the bacterial tryptophan RNA-binding attenuation protein (TRAP), which binds its target RNA sequence close to the transgene initiation codon. The rearrangement of the RNA secondary structure inhibits the translation initiation, preventing transgene expression without impacting viral titer yield [63]. However, additional studies demonstrated that the system’s repression efficacy was compromised when the viral RNA was aberrantly spliced in regions upstream of the TRAP binding sequence [64].

Recently, another silencing technology has been filed for patent (WO 2024/194651). This technology is based on replacing the promoter for the transcription of the viral genome that is going to be packaged. The expression of plasmids comprising the LVV production system is usually driven by RNA polymerase II (Pol II) promoters, commonly the strong CMV promoter. However, Pol II transcription usually terminates with a poly(A) tail, thereby directing the transcript into protein translation. To overcome the potential toxicity of therapeutic gene expression in the producer cell line, this Pol II is replaced by an RNA polymerase I (Pol I) promoter for the generation of the transfer genome RNA molecule. These transcripts are not modified by poly(A) tail addition, thereby ensuring that the generated transcript is available for packaging and not misdirected to the cell protein production machinery, risking incorrect splicing. This technology, where the Pol I promoter is combined with LTR sequences and the Pol I termination signal, provides an improved system with enhanced efficiency, reduced producer cell toxicity, and other issues associated with expression of the transgene [65]. Nevertheless, for complete silencing of the transgene in the producer cell, potential transcription from the internal promoter might also be considered.

#### 2.1.4. Splicing Escape

As previously indicated, the HIV-1 genome transcript undergoes several splicing events between its four donor (SD) and ten acceptor sites (SA) to create different transcripts that lead to several proteins required for virus formation [6]. The separation of the genome into the essential helpers for third-generation LVV development reduces the frequency of splicing sites within the transfer genome. Specifically, the Major Splicing Donor (MSD) is embedded in the packaging signal sequence, and the splicing acceptor 7 (SA7) is present in the *Env* element. Therefore, potential splicing events between those sites might lead to aberrant but packageable viral genome RNA variants, and, consequently, non-functional viral particles [66]. To give a solution, the implementation of mutations that ablated constitutive splice sites led to the activation of new cryptic sites, leading to unpredictable splicing events [54,67,68]. Recently, a novel technology was developed to avoid aberrant viral genome RNA generation through the mutation of the MSD and the adjacent cryptic SD (2KO genome) [64]. This double mutant increased the concentration of unspliced viral RNA (vRNA) generation. The addition of U1 snRNA enhancers into lentiviral vector production promotes the transcription of full-length viral RNA by its specific binding to packaging sequences that promote its processing and stability. Overall, LVV particles with full-length vRNA molecules devoid of spliced vRNAs are produced. This 2KO genome was combined with the previously mentioned TRAP system, which showed better LVV titers [64].

Additionally, splicing might also occur within the introns present on the vector payload. It is rather common to use internal promoters that contain intronic sequences to drive transgene expression in the transduced cells, as their presence can increase transcript levels as well as the efficiency of mRNA translation [69,70]. However, unwanted splicing of those sequences during vector assembly might decrease the expression of the transgene in the target cell. As a solution, with the aim of preserving a functional intron in the lentiviral vector, several groups reversed the orientation of the promoter and the GOI in the vector genome [71,72,73]. However, the transcript from the internal promoter led to an antisense transcript complementary to the full-length viral RNA, generating double-stranded RNA in the vector packaging cell line. These dsRNAs might then activate innate antiviral immune responses, including repression of mRNA translation by the protein kinase RNA-activated (PKR). Therefore, the inactivation of the PKR gene from the producer cell line restores lentiviral production by allowing substantial LVV titers when expressing reverse-oriented genes. Even if the integrity of the packaged or delivered viral genomes is not evaluated in these studies, a considerable improvement is expected due to the reduced recurrence of aberrant splicing events promoted by the presence of the intron in reverse configuration during transcript processing [72,73].

### 2.2. Optimizations in the Producer Cell Line

#### 2.2.1. Retro-Transduction

A key phenomenon worth studying involves the LVV-mediated transduction of producer cells during vector production. VSV-G pseudotyped lentiviral particles bud from the producer cell and remain in the cell culture media during part of the process, potentially transducing the producer cell line through LDLR (low-density lipoprotein receptor) interaction. This phenomenon is called retro-transduction or re-entry and has been reported to cause 70% vector yield loss due to producer cell transduction [74].

Several strategies have been employed to avoid re-entry of the viral vectors during production with the aim of recovering LVV yield. A study based on VSV-G pseudotyped lentiviral vectors centered on strategies avoiding the interaction of the viral VSV-G with the LDLR present on the cell membrane [75]. One of the strategies focused on LVV producer cell line (HEK293T) modification by knocking out the *LDLR* from the cells, and, thus, removing this entry pathway. This way, re-entry was reduced by up to 61%, although this reduction in LVV loss did not result in a yield titer increase. Evidence suggested that *LDLR* knockout was associated with altered cholesterol metabolism, leading to impaired LVV yield [75]. Conversely, other studies have documented an improvement in yield when *LDLR* was similarly knocked out when aiming to enhance LVV yield [76]. In this case, the observed increase in titer was explained by an alternative hypothesis suggesting that VSV-G and LDLR immature complexes might be retained between the ER and Golgi, rerouting them to degradation, hence hindering viral particle formation [77]. Additional strategies to avoid retro-transduction centered on viral VSV-G blockage with several molecules, such as soluble LDLR, specific CR3 domain of LDLR, or engineered RAP (receptor-associated protein) molecule [75]. Overall, the strategies showed contradictory results regarding the retro-transduction of the viral particles and the resulting LVV titers [75,76].

Recently, a promising approach has been developed to inhibit retro-transduction and, resultingly, increase LVV titer yields. This method is based on VSV-G conformational change at pH 6, which inhibits its interaction with LDLR and employs an LVV production method in which pH is shifted to pH 6.7–6.8 after transfection [78]. Results showed a seven-fold decrease in retro-transduction, leading to an almost two-fold yield increase in LVV. Crucially, pH-induced VSV-G conformational change is reversible, and an increase in pH transitions VSV-G back to a conformation allowing target-cell transduction [78].

#### 2.2.2. Engineering of the Producer Cell Line

For clinical and commercial applications, reaching high LVV production titers is essential, though challenging at times due to the length or complexity of the therapeutic gene. The transfection of LVV components into the cell line and the subsequent viral particle formation activate several restriction factors (RFs) in the producer cell that might compromise efficient lentiviral production. Therefore, multiple research groups have focused on identifying endogenous genes that could be modified to improve viral production.

Currently, the LVV production relies on the HEK293T cell line, a modified version of the parental HEK293 cell line that showed enhanced LVV titers through the integration of the SV40-derived T antigen [79]. Nonetheless, research on additional engineering of the HEK293T cell line as an LVV producer cell remains an underexplored area, potentially improved by uncovering correlations between endogenous genes and viral vector particle production yield. In this regard, CRISPR-Cas9-mediated genome-wide knockout (KO) screenings in HEK293T cells have enabled the identification of genes whose knockout might increase LVV titers. This way, several genes have been identified as associated with lipid metabolism involved in cell membrane homeostasis and innate immunity factors against pathogens [80]. Single-gene knockout of the genes GBP3 (guanylate-binding protein 3), BPIFC (BPI fold containing family C), NHLRC1 (NHL repeat-containing E3 ubiquitin protein ligase 1), LDAH (lipid droplet-associated hydrolase), and ZNF425 (zinc finger protein 425) performed in HEK293T resulted in LVV yield improvement. Subsequent multigene knockout studies resulted in triple knockout of GBP3, BPIFC, and LDAH as the optimal combination with approximately 8-fold LVV titer increase [80]. Similarly, a separate investigation also conducted a CRISPR-mediated KO study that led to the development of the cell line called CHEDAR (CRISPRed HEK293T to Disrupt Antiviral Response) by knocking out OAS1 (2′-5′-oligoadenylate synthetase 1), LDLR, and PKR, which significantly increased LVV titers [76]. These genes are restriction factors that inhibit specific steps of the LVV production process. For instance, OAS1 is activated by double-stranded RNA, leading to RNase L-mediated degradation of the viral RNA. Similarly, PKR is activated by viral sequence TAR or double-stranded RNA, resulting in translation inhibition. This dsRNA-induced activation leads to an additional titer increase when the transgene is reverse-oriented, as previously mentioned [72,73]. As for LDLR, the increment in titer observed in its absence has been attributed either to the avoidance of retro-transduction or to intracellular mechanisms leading to degradation of immature VSV-G-LDLR complexes [77]. Further enhancements in CHEDAR cell line LVV production yield were achieved by overexpressing elongation factors SPT4 and SPT5 during packaging [76].

As stated earlier, third-generation LVVs are devoid of accessory proteins, including Vif, which counteract the proteosomal degradation induced by APOBEC3G [42]. Hence, the absence of the Vif protein in third-generation LVV renders them susceptible to APOBEC, a family of DNA cytidine deaminase proteins with various members, from which APOBEC3B and APOBEC3G are involved in the restriction of lentiviruses. Indeed, APOBEC expression during lentiviral vector production might lead to its co-packaging into LVV particles through interaction with NC and viral RNA. When those LVVs transduce a new cell line, the APOBEC enzymatic activity is capable of mutating the DNA by cytidine deamination during reverse transcription. A study was conducted in which APOBEC3B knockout in LVV-producing HEK293T cells resulted in a slight but significant decrease in mutational load in the LVV genome and increased functional activity of the resultant CAR T cell product [23,81,82]. However, low levels of APOBEC were packaged into LVV particles, leading to low-frequency mutations; thus, its real impact remains to be demonstrated and requires evaluating if the potential rate of mutations is minimally concerning.

The producer cell endogenous gene modifications mentioned were primarily focused on enhancing LVV manufacturing yields. However, the gene expression profile of the producer cell may influence other aspects of the LVV. For instance, LVVs acquire their envelope during the budding process from the producer cell; hence, the viral particle incorporates producer-cell-derived membrane proteins as integral parts of its mature envelopes [83]. The presence of producer-cell-derived proteins in viral envelopes may compromise target cell transduction due to the host organism’s immunogenic response, specifically for in vivo delivery approaches. In fact, producer cell-derived polymorphic class-I major histocompatibility complexes (MHC-I) are incorporated into the LVV surface and trigger allogeneic T-cell responses. To prevent this, MHC-I-negative producer cells were generated by genetic disruption of β-2 microglobulin (B2M), which is required for MHC-I expression on the cell membrane. This cell line produced MHC-I-free LVVs with the same infectivity but lower immunogenicity than conventional LVVs [84]. However, additional preclinical studies have related the reported mild acute toxicity and low efficacy of systemically administered LVVs to fast circulation clearance by phagocytes [85]. The strategy proposed to counteract LVV capture by phagocytes consists of CD47 overexpression in the LVV envelope, a natural phagocytosis inhibitor. For that, CD47 is overexpressed in the producer cell, which has no impact on the manufacturing yield nor the infectivity of the generated LVV. Instead, CD47 presence in the viral envelope decreases LVV intake by phagocytes, augmenting target cell transduction efficiency and reducing the immunogenic response [85].

#### 2.2.3. Packaging/Stable Producer Cell Lines

The standard method for the large-scale manufacturing of GMP-grade lentiviral vectors involves multiple steps. Upstream processing (USP) starts with the culture and expansion of the LVV-producing cell line HEK293T. For vector production, the cells are transfected with the helper and transgene plasmids by means of a transfection reagent. The USP ends with the viral supernatant harvesting, where the downstream phase (DSP) starts. Bulk harvest is purified, concentrated, and sterile filtered to subsequently formulate in the specific matrix. Eventually, the final product is characterized by functional or physical titer analysis, along with an exhaustive purity and potency evaluation. This procedure is effective for the current market demand but requires optimization to enhance reproducibility and improve cost-efficiency.

LVV manufacture through transient transfection is a suitable method for small-scale production, although it poses some challenges when scaling up. The transfection step requires expensive raw materials, mainly DNA and transfection reagents, and allows limited control of the process when transitioning into industrial-scale production [86,87]. The sensitivity of the transfection reagents, such as the PEI, to ionic strength, ratio to DNA, or incubation leads to variable complex formation that may limit the reproducibility of the system [88]. Moreover, residual plasmid DNA remaining after the transfection step must be cleared from the LVV product to avoid its transfer to patient cells during vector administration [86].

To address these challenges, several groups have developed LVV packaging and producer stable cell lines, where the three helper genes are integrated in the cell genome alone or along with the transgene of interest, respectively [89,90,91]. Historically, the first cell lines generated were packaging cell lines pseudotyped with the HIV-1 envelope [92,93,94]. The helpers were generally expressed in a constitutive manner, and the cell line required vector genome transfection for LVV production, which still represents a reproducibility hurdle. Later, the HIV envelope was exchanged for several other envelope proteins, with VSV-G as the preferred choice due to its wide tropism and stability during LVV manufacturing steps [17,95,96]. Therefore, the following producer stable and packaging cell lines were mainly pseudotyped with VSV-G. Nevertheless, the VSV-G is cytotoxic [97]. Thus, some groups preferred a non-cytotoxic approach and implemented some other pseudotypes, such as RD114-TR [98,99], Cocal [100], MLV [87], or SVGmu [101]. Although VSV-G pseudotyped virus yields are usually higher [98], differently pseudotyped cell lines, such as the one with Cocal envelope, also showed substantial titers and superior infectivity/potency in HSC or T cells, greater than VSV-G [100]. Overall, VSV-G is the preferred choice for the viral envelope, although its cytotoxic activity requires a control system. Additionally, HIV protease expressed from Gag-Pol polyprotein has also been reported to induce cytotoxic effects, which further challenges stable cell line generation [87]. The implementation of an inducible promoter system has been the strategy of choice to control cytotoxicity. The tetracycline-regulated promoter system is the predominant choice for inducible packaging and stable cell lines [89,91,102], although cumato- and ecdysone-regulated systems are also used [90,103,104]. By just adding or removing these inducers from the culture media, lentiviral production is induced by a plasmid transfection-free method. These molecules are expected to be efficaciously removed by standard downstream processing platforms established for LVV purification and concentration. Although the generation of packaging and stable producer cell lines is arduous to accomplish, multiple benefits are achieved, particularly an increase in reproducibility and a reduction in costs associated with plasmid and transfection reagents.

## 3. New Opportunities for LVV

Nowadays, new genetic drugs and delivery vehicles are constantly being developed, broadening the application of these tools in gene and cell therapies. The genetic drug is directly responsible for the therapeutic effect on the target cell and might be introduced in the form of DNA, RNA, or protein. Meanwhile, delivery vehicles refer to the vector that carries the genetic drug and can be viral, non-viral, or a physical transfer method. These components are combined to adapt to the treatment requirements of each disease and give a robust, fast, and safe therapeutic effect. However, given the diverse requirements of each disease, there is no one-size-fits-all solution. An assessment of the advantages and drawbacks of each combination is essential to select the most suitable option accordingly. With lentiviral vectors as the main focus of this review, new engineered modalities of these vectors will be presented as alternative options to cover the current gaps in the field.

### 3.1. LVV Pseudotyping for In Vivo Administration

The clinical progress of cell and gene therapy owes its success to the increasing understanding of the delivery vectors used as carriers of the desired gene. Among the multiple types of viruses tested in preclinical and clinical phases, two main vector types emerged: adeno-associated vectors (AAVs) and lentiviral vectors (LVVs) [105]. Traditionally, in vivo cell modification has been restricted to AAV, while ex vivo approaches have relied on LVV. However, the field is constantly developing to address the remaining challenges; hence, this division is becoming fuzzier. In vivo therapies are an appealing option due to the direct administration of the gene delivery vector into the patient to reach a specific tissue, which simplifies treatment logistics and costs associated with the cell processing step performed for ex vivo treatments. These kinds of therapies leverage advances in vector engineering and delivery methods to enhance success rates while considerably reducing treatment costs.

Although the existing lentiviral treatments are currently ex vivo, in vivo therapies are now gaining traction since lentiviruses provide the advantage of an intrinsic low immunogenicity in the general human population with a reduced capacity to induce inflammation and innate immune responses [106,107,108,109]. Therefore, minimizing off-target events is crucial and is achieved, for instance, through specific pseudotyping of the virus. For ex vivo LVV-based therapies, no specific tropism is required for the vector, since the target cells are isolated before transduction. Therefore, pan-tropic envelope proteins such as VSV-G represent a suitable choice for pseudotyping the vector, as they permit the transduction of a wide range of cell types through their interaction with the ubiquitous LDL-R of the cell membrane. Moreover, VSV-G pseudotyped vectors possess favorable features for the different steps of their manufacturing process, such as an increased stability as well as a high resistance to freeze–thaw and harsh purification methods [17,95,96]. Nevertheless, other heterologous envelopes, such as the envelope of Baboon endogenous virus (BaEV) as well as feline endogenous retrovirus (RD114), have been shown to facilitate ex vivo HSC transduction through recognition of the sodium-dependent neutral amino acid transporter (ASCT-2) present on CD34+ T and B cells, and additionally, ASCT-1 in the case of BaEv [110]. Also, BaEV pseudotyped viruses are not neutralized by exposure to human serum complement, which might be advantageous for potential in vivo approaches [110]. Engineered options are also available, such as the chimeric versions of Gibbon Ape Leukemia Virus (GaLV) and RD114 (e.g., GaLV-TR and RD114-TR, among others), which showed increased titers, stability, and infectivity [111,112].

Regarding in vivo approaches, when the natural biodistribution that follows the systemic administration of vectors is favorable, such as in liver-targeting therapies, LVVs pseudotyped with the VSV-G have proved to be successful [113,114]. Alternatively, they can be administered locally, for example, via colorectal administration [115]. Nevertheless, some studies indicate that VSV-G might be inactivated by human serum complement [116], thus compromising in vivo performance. In this context, Cocal envelope appears as a suitable alternative as it recognizes LDL-R just like VSV-G and is resistant to human serum complement [100,117]. The presence of multiple alternatives facilitates the in vivo delivery tailoring for each individual according to its pre-existing condition, as well as enabling combinations to avoid resistance in cases of re-administration. However, for in vivo cell modification of tissues other than the liver, high specificity of the target cell is essential to avoid off-target events. Therefore, several other envelopes have been tested to direct the tropism of LVV. For instance, LVV enveloped with muscle-specific cell fusogens myomaker and myomerger have been shown to efficiently and specifically transduce skeletal muscle in vivo, in the dystrophic myopathies, and during muscle overload. Indeed, this pseudotyping technology demonstrated therapeutic utility for Duchenne muscular dystrophy, for example, a genetic muscle disease resulting in chronic cycles of muscle injury and regeneration [118]. Additionally, the rabies virus (RV) glycoprotein specifically targets neurons and has demonstrated efficient transduction into spinal cord neurons after muscle injection in rats [119]. Further efforts for the development of target-specific LVV involve protein engineering. These engineered proteins comprise a target-specific ligand directing cell binding and a mutated viral glycoprotein enabling membrane fusion and viral entry. Commonly used ligands include single-chain variable fragments (scFv), designed ankyrin repeat proteins (DARPins), or cytokines, among others. As a viral glycoprotein, the measles virus hemagglutinin (MV-H) protein can be mutated to abolish its natural tropism (SLAMF1 and NECTIN4 receptors). Studies where MH-V is combined with specific scFv or DARPins have demonstrated high tropism for potential in vivo CAR-T therapies [120]. Limitations regarding low functional titers and pre-existing MV neutralizing antibodies in the host organism led to the development of alternative pseudotypes. For instance, a recent study generated LVV pseudotyped with a chimeric measles virus/Dolphin Morbillivirus (DMV) envelope, which showed resistance to potential pre-existing anti-MV immunity. They further revealed that camelid-derived single-domain antibodies (nanobodies or VHHs) are more successful at targeting domains for MV-H fusions [121]. Many other viral envelopes have been mutated and tested as fusogens, such as the Nipah virus, Sindbis virus, or the common VSV, although the latter’s pH dependency might be a constraint for efficient target cell transduction [120]. Despite these promising strategies, it is only recently that the first in vivo-based therapies reached clinical phases. Examples of ongoing in vivo clinical trials include ESO-T01 (EsoBiotec SA, Mont-Saint-Guibert, Belgium), a BCMA-targeting CAR for relapsed/refractory multiple myeloma; UB-VV400 (Umoja Biopharma, Seattle, WA, USA), which consists of a CD22 CAR for relapsed/refractory diffuse large B cell lymphoma; and UB-VV111 (Umoja Biopharma, Seattle, WA, USA), a lentivirus carrying CD19 CAR for relapsed/refractory chronic lymphocytic leukemia and other B-cell malignancies; all of them in phase I [122].

### 3.2. Integrase Deficient Lentiviral Vectors (IDLVs) and Their Application in Vaccinology

While integrative viral vectors are essential to provide stable transgene expression in the transduced cells, they also raise concerns about genotoxicity, primarily due to the risk of insertional mutagenesis. Despite the remarkable progress on the safety profile of LVVs, secondary primary malignancies (SPMs) have been reported in commercialized LVV-based CAR-T therapy-treated patients, although the frequency of occurrence is low [123,124,125,126,127]. Despite the lack of clear evidence about the direct correlation between the CAR-T treatment and the SPM emergence [128], these events prompted the FDA requirement to add boxed warnings to the labels of six CAR-T therapies, indicating the risk of secondary cancer. Integration site profiling performed in several clinical trials with SIN-LVVs has demonstrated a wide integration area leading to polyclonal modified cell populations with no clonal outgrowth or malignant transformation [129,130]. Indeed, there are certain cases of clonal expansion that were non-malignant and still resulted in disease remission [131,132]. Additionally, the clinical background of each patient concerning their prior malignancy, as well as the initially received first-line treatments, such as chemotherapy or radiotherapy, increases the SPM development probability. The overall low incidence of SPM provides reassurance of the safety profile of available CAR-T cells engineered by LVVs, although the inherent genotoxic risk requires ongoing monitoring, reduction to the extent possible, and thorough risk-benefit assessments.

Despite the extensive information demonstrating the safety profile of integrative lentiviral vectors, the prevailing concern of potential genotoxicity due to insertion prompted the development of integrase-deficient lentiviral vectors (IDLV). IDLVs result from leveraging the circular forms with 1- or 2-LTR generated as non-integrated double-stranded DNA products from reverse transcription of the viral genome. The 1-LTR DNA circles are formed by homologous recombination between the two viral LTRs, while 2-LTR containing circular viral DNA is produced by non-homologous end joining [133,134]. Over recent years, these circular DNA forms have been shown to be transcriptionally active, so several groups have employed them for gene expression in the absence of integration with gene therapy and immunization purposes [11]. The integration process is mediated by the integrase protein, encoded by the *pol* gene. This protein is responsible for nuclear import of the preintegration complex (PIC) and plays a role in the reverse transcription of the viral RNA genome [135,136]. Therefore, the deletion of the whole integrase gene is not feasible, although specific point mutations in the integrase gene are sufficient to interrupt its normal function. This can be effectively attained by introducing mutations in the IN protein of the vector and/or at the DNA attachment site (att) on the U3 region of the LTR, thus preventing integration while maintaining the generation of the circular forms [137]. Integrase contains a core DDE amino acid sequence motif called the catalytic triad, located at positions D64, D116, and E152, absolutely required for integration [138]. Point mutations in the catalytic site of integrase at those positions have been shown to specifically inhibit integration of viral DNA into the host genome [139,140,141,142]. So, circular DNA episomes resulting from the absence of integration offer a sustained transgene expression, particularly in non-dividing cells [143,144,145,146,147,148], since in proliferating cells, proviral burden is diluted with cell division due to lack of origin of replication (ORI). Notably, a patent was granted for a technology where non-integrative and autoreplicative DNA episomes are established by IDLV transduction. These episomes contain a scaffold/matrix-associated region (S/MAR) element for their anchoring to the nucleus along with an origin of replication (ORI) for the autonomous replication of the episome upon cell division [149]. Although this technology holds significant potential, further development is required to increase the episome establishment frequency, as well as the analytical tools for circular episome detection, mainly in polyclonal cell populations.

One of the primary areas where IDLVs are utilized is the field of vaccinology. Viral vector-based vaccination leverages its ability to deliver antigens or activation signals directly to antigen-presenting cells (APCs), thereby promoting T lymphocyte activation for effective immunization [150,151,152]. In this context, LVV is known to be an efficient vehicle for genetic modification of dendritic cells (DCs) in vitro [153], although it might have off-target effects when administered in vivo. With the goal of minimizing off-target effects and improving safety, multiple strategies based on alternative engineered glycoproteins have been described to redirect LVV to APC-specific cell surface receptors, including pseudotyping with nanobodies or chimeric proteins targeting DCs [154,155,156]. There are several preclinical studies for prophylactic LVV-based therapies, such as vaccination based on IDLV administration encoding specific epitopes, inducing immunogenic response against Zika, SARS-CoV-2, or influenza virus [157,158,159]. Apart from cellular and humoral immunity, IDLVs have also been proven successful for sustaining anti-tumor immunity. Leveraging the presence of tumor-associated antigens (TAA) expressed by cancer cells, several anti-tumoral vaccines are being developed [153]. For instance, an onco-therapeutic vaccine candidate against human papilloma virus (HPV)-induced tumors has been developed, called Lenti-HPV-07. This vaccine is based on an IDLV encoding HPV oncoproteins and has obtained successful preclinical results with complete eradication of tumors and long-lasting memory response protecting from tumor recurrence [160].

### 3.3. Reverse Transcriptase Deficient LVV for RNA Delivery

Although IDLVs establish a non-integrative DNA episome in the target cell, there is evidence of possible residual integration of the lentiviral genome due to DNA damage and cellular metabolism that might end in non-LTR-mediated genomic integration by NHEJ [137,161]. As an alternative strategy for inherently transient gene expression, genetic material can be delivered as an RNA molecule. Several applications require transient expression of the desired gene, such as exogenous transcription factor expression for cell reprogramming or genome editing through recombinases or nucleases. Therefore, delivery of RNA emerges as a transient expression platform, where potential integration is avoided. Within this framework, non-viral vectors are often the preferred delivery vehicles for RNA-based therapeutics due to their safety profile and ease of manufacturing [162,163,164]. Nevertheless, some aspects continue to compromise their efficiency, such as cell uptake or endosome escape [162,165].

In this context, the intrinsic nature of lentiviral vectors to package ssRNA molecules renders them ideal candidates for RNA delivery. To recall, when VSV-G pseudotyped LVV transduces the target cell, the viral RNA is reverse transcribed into DNA for subsequent integration into the host cell genome. Thus, to guarantee immediate RNA translation in the cytoplasm, several strategies have been developed for reverse transcription step suppression. Among them, mutations in the catalytic domain of the RT protein, mutations in the PBS sequence of the vector genome, or nucleoside analogs to block reverse transcription through incorporation of false nucleosides [166,167]. This way, as the plus-stranded vector RNA contains a 5′cap structure and a 3′ poly(A) tail, it may serve as an immediate template for de novo translation of any protein of interest in the cytoplasm. Nevertheless, LVV genome direct translation is rather inefficient, most probably due to the large 5′ untranslated region. Accordingly, a strategy to facilitate cap-independent translation initiation was developed by introducing an internal ribosomal entry site (IRES) for the expression of the gene of interest [168]. Reverse transcription was inhibited by enzymatic inactivation of the RT, mutating its catalytic site from YMDD to YMVV. With the very same objective of tackling the weak cap-dependent translation, a different study positioned the HIV-1 material from the 5′ site of the vector genome to downstream of the 3′LTR [55,169]. This displacement allowed ribosomal entry from the m7G 5′ cap and hence the gene of interest translation in a cap-dependent way. Correspondingly, the RT protein was engineered, and additionally the PBS sequence from the transgene was removed for total inhibition of reverse transcription. Further attempts to enhance LVVs for non-viral mRNA delivery include the development of a dimerization-independent RNA recruitment technology [170]. This technology is based on the incorporation of the bacteriophage MS2-Coat protein into LVV capsids and engineering the MS2 RNA stem loop into LV genomic RNA. This recruitment system allows the packaging of 5–6 copies of non-viral RNA molecules in a single particle, offering a rapid and transient expression of the RNA upon cell entry. These engineered LVV particles are devoid of IN or RT; thus, no integration nor reverse transcription happens.

### 3.4. Lentivirus Derived Nanoparticles (LVNPs) for Gene Editing

Progress in advancing gene therapy technologies includes the discovery and development of site-specific nucleases for gene editing, such as zinc finger nucleases (ZFN), transcription activator-like effector nucleases (TALEN), and the clustered regularly interspaced short palindromic repeats (CRISPR) and CRISPR-associated protein (Cas) system (CRISPR-Cas). These gene editing tools induce double-strand breaks (DSB) in the target genome that are usually corrected by an error-prone non-homologous end joining (NHEJ) mechanism, which results in deletions or insertions (InDels) in the genome. Alternatively, if a template DNA molecule is provided, the cell will activate a homology-directed repair (HDR) mechanism, which results in an error-free replacement of the target sequence [171,172,173,174]. ZFN and TALEN arise from the combination of site-specific nucleases (*FokI*) with programmable DNA-binding protein domains. They function as dimers, where each monomer binds to opposite strands of the target genome, leading to dimerization of the nuclease domain and DSB with high specificity and fidelity [175]. The CRISPR-Cas system emerged as an innovative platform for both genome and epigenome editing in cells. Among various subtypes, the CRISPR-Cas9 system is the most studied and developed one and includes several components for genome editing [176]. Briefly, the system is composed of a Cas9 protein, which is an endonuclease enzyme with two catalytic domains that perform a double-strand break (DSB) in the genome, and a synthetic guide RNA (sgRNA) that combines a tracrRNA to bind the Cas9 protein and crRNA, which binds the target DNA [171,177]. Thus, the target site recognition of this system is based on a specific RNA sequence, as opposed to ZFN or TALEN, which rely on protein binding to DNA. This difference streamlines gene editing design and implementation, as RNA molecule synthesis is easier and more straightforward than engineering proteins.

So, for successful therapeutic gene editing, the selection and development of the optimal gene editing tool is required, along with the specific delivery mechanism. There are several modalities for the delivery of these elements regarding the cargo and the delivery vehicle. The nuclease might be delivered in the form of a gene expression DNA cassette, RNA molecule, or directly as a protein. Moreover, a repair template might be included to promote HDR for gene correction and gene addition approaches. As genome modification requires a single-shot performance with minimal off-target events, transient expression of the elements is preferred in the form of RNA or protein delivery. The safest choice by default is administering the protein itself and not the protein production source; thus, the genome-modifying activity will only last until the protein is diluted and degraded.

Delivery methods include physical approaches, non-viral or viral vectors, and the choice of the method will determine the form in which the cargo is delivered, or vice versa. Physical delivery methods such as microinjection or electroporation have proven to be efficient for the transfer of the cargo in all forms, although they only permit ex vivo administration [178,179,180]. Instead, non-viral vectors like LNPs have high packaging capacity and can be used for ex vivo and in vivo applications. Indeed, recently, a neonate diagnosed with severe carbamoyl-phosphate synthetase 1 deficiency was treated with LNP-delivered base-editing therapy, with initial successful outcomes, although safety and efficacy assessment of the treatment will require longer follow-up [181]. However, these delivery methods show difficulties for nuclear transfer of the elements and are therefore generally not considered as the first choice [182]. Viral delivery of the CRISPR-Cas9 system can encounter some challenges, too. Firstly, the large size of the Cas9 gene (around 4.2 kb) can limit packaging capacity within the AAV particle. As a solution, a split-intein Cas9 system separated into two AAV cassettes was developed, although it requires the production and co-transduction of two viral particles [183]. Secondly, continuous expression of Cas9 in transduced cells can increase the risk of off-target effects, as the enzyme may bind to unintended DNA sequences. Finally, the need to deliver Cas9 alongside the sgRNA, which may be packaged separately in some cases, adds complexity and can further reduce efficiency. Therefore, viral vectors have been the carrier of choice for the delivery of a repair template for HDR, aiming at gene correction or addition. So far, recombinant AAV2/6 has been the preferred template vector [184,185,186]. Nevertheless, recent studies have shown that unexpected load and persistence of AAV genomes and fragments can trigger sustained p53-mediated DNA damage response (DDR) by ITR-related recruitments in HSPCs [187]. Alternatively, LVVs have also made their way into the repair template delivery, specifically in IDLV format, which allows the GOI expression from a non-integrative DNA episome. Comparison of AAV- and LVV-based repair template delivery suggests IDLV as a lower cytotoxic and genotoxic choice, with lower DNA load and faster decay in transduced cells, but high knock-in efficiency [187].

Additionally, the ability of lentiviral vectors to package non-viral proteins has led to exploiting them as all-in-one particles for the delivery of the specific nuclease that induces the DSB, along with an sgRNA molecule for Cas9 targeting or, optionally, a donor sequence for the HDR of the targeted genomic region. The non-viral protein packaging is based on fusion proteins of Gag and Gag-Pol polyproteins that will release the fused nuclease thanks to protease cutting. Initial strategies relied on fusing the protein of interest to Vpr [188]; however, the few copies of Vpr incorporated into proviral particles, combined with its potential toxicity to target cells, made it an unsuitable option. Alternatively, fusion proteins were developed as part of Gag/Gag-Pol polypeptides, which are embedded in the plasma membrane of the proviral particle. Maturation of the viral particle includes cleavage of the polypeptides by the viral protease; hence, the fusion of proteins into Gag or Gag-Pol polypeptides leverages this process to package non-viral proteins into the viral particle [189]. The protein is usually fused to the N-terminal of the Gag protein, separated by a phospholipase C-δ1 pleckstrin homology (PH) domain for membrane harboring [189,190,191,192]. The maturation of the virion leads to the cleavage and release of the protein in the target cell. This strategy has allowed the packaging of ZFN [190,191,192], piggyBac transposase [193,194], or even CRISPR-Cas9 element [189,195,196]. For instance, all-in-one particles containing RNP, including the Cas9 and sgRNA, were generated with a high on/off-target editing ratio [189]. This approach additionally allows the delivery of new CRISPR modalities, such as base-editing or prime-editing systems for the correction of single nucleotides in target sequences. The most well-known base-editing systems are the cytosine base editor (CBE) and adenosine base editor (ABE), which are based on single-strand break (SSB) performing nickase Cas9 (nCas9) together with a base-editing enzyme [197,198]. Meanwhile, prime-editing systems perform corrections of pyrimidine to purine nucleotides. Basically, they are based on an nCas9 fused to a reverse transcriptase, which will use as a primer for reverse transcription the DNA strand accessible after Cas9 cleavage [199]. Ultimately, the delivery mechanism for HDR-directed repair may involve LVNP packaging of a viral RNA molecule that, upon cell transduction, is reverse transcribed to a DNA molecule that serves as a repair template. However, these particles are yet unsuitable for HDR-based gene editing. So, for cases in which large exogenous gene insertion is desired, the template DNA is usually provided by a second IDLV, and a double transduction of the cells is performed.

Overall, the field continues to actively work on expanding the range of applications for LVV-based therapies, and these technologies serve as a clear example of that progress. The suitability of each technology needs to be evaluated for optimal treatment options for each disease, based on factors such as the type of administration and whether a transient or long-lasting therapeutic effect is required. Regardless, the field is advancing at a ceaseless pace, with increasingly promising results supporting the potential of LVV as multi-functional delivery tools. Nonetheless, continued development is necessary to enhance efficacy and efficiency while preserving safety.

## Abbreviations

AAV	Adeno-Associated Virus
ABE	Adenosine Base Editor
ALDP	Adenoleukodistrophy Protein
APC	Antigen-Presenting Cells
APOBEC	Apolipoprotein B MRNA Editing Enzyme
ASCT	Alanine, Serine, Cysteine Transporter
ATMP	Advanced Therapy Medicinal Product
Att	DNA Attachment Site
B2M	Β-2 Microglobulin
BaEv	Baboon Endogenous Virus
BPIFC	Bpi Fold-Containing Family C
CA	Capsid
CALD	Cerebral Adrenoleukodystrophy
CAR	Chimeric Antigen Receptor
CBE	Cytosine Base Editor
CDMO	Contract Development And Manufacturing Organization
CRISPR	Clustered Regularly Interspaced Short Palindromic Repeats
CTE	Constitutive Transport Element
DARPins	Designed Ankyrin Repeat Proteins
DC	Dendritic Cells
DDR	DNA Damage Responses
DIS	Dimerization Initiation Site
DMV	Dolphin Morbillivirus
DSB	Double-Strand Break
DSP	Downstream Processing
GaLV	Gibbon Ape Leukemia Virus
GBP3	Guanylate-Binding Protein 3
GMP	Good Manufacturing Practices
GOI	Gene Of Interest
HDR	Homology-Directed Repair
HIV-1	Human Immunodeficiency Virus Type 1
HPV	Human Papillomavirus
HSC	Hematopoietic Stem Cell
HSPC	Hematopoietic Stem And Progenitor Cell
IDLV	Integrase Deficient Lentiviral Vector
IN	Integrase
IRES	Internal Ribosome Entry Site
ITR	Inverted Terminal Repeat
LDAH	Lipid Droplet-Associated Hydrolase
LDLR	Low-Density Lipoprotein Receptor
LNP	Lipid Nanoparticle
LTR	Long Terminal Repeats
LVNP	Lentivirus-Derived Nanoparticles
LVV	Lentiviral Vectors
MA	Matrix Protein
MHC-1	Major Histocompatibility Complexes Class 1
MLD	Metachromatic Leukodystrophy
MPMV	Mason-Pfizer Monkey Virus
MSD	Major Splicing Donor
MV-H	Measles Virus Hemagglutinin Protein
NC	Nucleocapsid
Nef	Negative Factor
NHEJ	Non-Homologous End Joining
NHLRC1	NHL Repeat-Containing E3 Ubiquitin Protein Ligase 1
NPC	Nuclear Pore Complexes
OAS1	2′-5′-Oligoadenylate Synthetase 1
ORF	Open Reading Frame
ORI	Origin Of Replication
PBS	Primer Binding Site
PH	Pleckstrin Homology
PIC	Preintegration Complex
PKR	Protein Kinase RNA-Activated
PPT	Polypurine Tract
PR	Protease
RCL	Replication Competent Lentivirus
RD114	Feline Endogenous Retrovirus
Rev	Regulator Of Expression Of Virion Proteins
RF	Restriction Factor
RRE	Rev Responsive Element
RT	Reverse Transcriptase
RTE	RNA Transport Element
RV	Rabies Virus
S/MAR	Scaffold/Matrix Associated Region
SA	Splicing Acceptor
scFv	Single-Chain Variable Fragments
SD	Splicing Donor
SIN-LVV	Self-Inactivating Lentiviral Vectors
SPM	Secondary Primary Malignancies
SRV-1	Simian Retrovirus Type 1
SU	Surface Glycoprotein
TAA	Tumor-Associated Antigens
TALEN	Transcription Activator-Like Effector Nucleases
TAR	Transactivation-Reponse Element
Tat	Trans-Activator Of Transcription
TM	Transmembrane Glycoprotein
TRAP	Tryptophan RNA-Binding Attenuation Protein
USP	Upstream Processing
VHH	Variable Heavy Domain Of Heavy Chain
Vif	Virion Infectivity Factor
Vpr	Viral Protein R
Vpu	Vira Protein U
VSV-G	Vesicular Stomatitis Virus Glycoprotein
WHX	Woodchuck Hepatitis Virus X Protein
WPRE	Woodchuck Hepatitis Virus Posttranscriptional Regulatory Element
ZFN	Zinc Finger Nuclease
ZNF425	Zinc Finger Protein 425
